# Congenital Genitourinary Abnormalities Associated With Prenatal Drug Exposure

**DOI:** 10.7759/cureus.117387

**Published:** 2026-10-03

**Authors:** Lisa B Shields, Kahir Jawad, Corey Moran, Eran Rosenberg

**Affiliations:** 1 Norton Neuroscience Institute, Norton Healthcare, Louisville, USA; 2 Norton Research Institute, Norton Healthcare, Louisville, USA; 3 Pediatric Urology Department, Norton Healthcare, Louisville, USA

**Keywords:** congenital, drug use, genitourinary abnormality, obstetrics, pediatrics, pediatric urology, prenatal

## Abstract

Background/objectives: Prenatal drug exposure poses a public health dilemma and adversely affects numerous neonatal body systems. Certain drugs during pregnancy may be associated with genitourinary (GU) abnormalities in the neonate, including opiates, cannabinoids, tobacco, alcohol, and psychostimulants. We sought to determine the incidence and characteristics of GU abnormalities in infants who were exposed to maternal drug use during pregnancy.

Methods: We identified patients with GU anomalies younger than one year of age who were exposed to maternal prenatal drug use over an 11-year period (January 1, 2014-December 31, 2024). We compared patients with GU abnormalities who were and were not exposed to maternal drug use. GU abnormalities concurrent with other congenital system abnormalities were also assessed.

Results: GU congenital abnormalities were identified in 18,467 (9.1%) of newborns, with 1,076 (5.8%) of these infants prenatally exposed to drugs. Infants prenatally exposed to opioids were 64% more likely to have GU congenital anomalies compared to those without opioid exposure (p < 0.0001). Infants prenatally exposed to cocaine were 59% more likely to have GU congenital anomalies compared to those without cocaine exposure (p = 0.034). Infants prenatally exposed to alcohol were 53% more likely to have GU congenital anomalies compared to those without alcohol (p = 0.043).

Conclusion: Physicians, parents, and other caregivers should be cognizant of the association between GU abnormalities and maternal drug use during pregnancy. Further research is necessary to determine the specific drugs and toxicities associated with GU abnormalities following prenatal drug exposure.

## Introduction

In 2021, approximately 400,000 (11%) births in the United States were affected by prenatal exposure to alcohol, and roughly 290,000 (8%) births were impacted by prenatal exposure to illicit drugs [1,2]. Between 2010 and 2017, the number of women with opioid-related diagnoses at delivery hospitalization increased by 131%, with our state of Kentucky having the fourth-highest maternal opioid-related diagnosis (MOD) rate (23.4 per 1,000 delivery hospitalizations) in the United States [3,4]. Kentucky's high MOD rate reflects its elevated opioid overdose mortality rate compared to the rest of the nation. The estimated opioid use disorder prevalence in Kentucky was 5.5% and 5.9% for 2018 and 2019, respectively, ranging from 1.3% to 17.7% across Kentucky counties [5]. Our state's increase in drug-overdose deaths during the start of the COVID-19 pandemic was the second-highest percentage increase in the United States.

Most drugs of abuse can cross the placenta and negatively impact fetal brain development [6,7]. Prenatal drug exposure to marijuana, cocaine, methadone, and heroin reportedly accelerates normal fetal brain maturation, which is accompanied by poorer 12-month infant outcomes [5]. Prenatal opioid use is associated with numerous adverse health outcomes, including preterm birth, stillbirth, small for gestational age, low birth weight, sudden infant death, and neonatal abstinence syndrome (NAS) [3,7,8]. Tobacco also poses several risks to the neonate, such as preterm birth, low birth weight, and sudden infant death syndrome, and psychostimulants may be associated with preterm labor and cardiac/cardiovascular/cranial abnormalities [3,7]. Alcohol may increase the risk of miscarriage, preterm birth, stillbirth, sudden infant death syndrome, and fetal alcohol spectrum disorder [3,7,9,10]. Cannabis may increase the likelihood of a low birth weight, preterm delivery, and neonatal intensive care unit admission. While the literature has addressed the adverse effects of alcohol and presription/illicit drugs on the developing fetus, few studies have discussed their ramifications on genitourinary (GU) development.

A large series of patients with GU abnormalities associated with prenatal drug exposure is lacking in the literature. In this report, we describe infants with GU abnormalities due to maternal drug use during pregnancy. The specific GU malformations, as well as the particular maternal drugs, are presented. We also highlight the other congenital system abnormalities that co-exist with GU abnormalities in infants who were exposed to prenatal drugs. The primary objective of this study was to assess infants younger than one year of age born with GU abnormalities due to maternal drug use during pregnancy who were evaluated by a pediatric urologist at a metropolitan community hospital over an 11-year period (January 1, 2014-December 31, 2024).

## Materials and methods

Under an Institutional Review Board (IRB)-approved protocol and according to the Declaration of Helsinki, we performed an 11-year (January 1, 2014-December 31, 2024) retrospective analysis of patients with congenital GU abnormalities associated with maternal drug use during pregnancy. All patients were younger than one year of age at initial presentation at our Institution. Study inclusion and exclusion criteria were used to create a structured query language (SQL) query that identified patients within our electronic health record (EHR) system. ICD-10 codes were stored as diagnoses in patients' charts and were used to identify individuals with various congenital abnormalities and drug exposure diagnoses. The following ICD-10 GU diagnostic codes were included: genital (Q50-Q56) and urinary (Q60-Q64) (Table 1). All patients had an in-person encounter (outpatient or inpatient setting). Patients who were older than one year of age were excluded.

**Table 1:** Congenital Genitourinary Abnormalities Associated With Prenatal Maternal Drug Use by ICD-10 Codes at Our Institution * Not a count of distinct patients; one patient may have had multiple International Classification of Diseases, 10th Revision (ICD-10) code indicators, indicating one or more corresponding congenital abnormality diagnoses. ** The most common conditions with the ICD-10 code Q55 included "other congenital malformations of the penis", congenital torsion of the penis, and hidden penis.

Genitourinary Abnormalities	ICD-10 Codes	Number of ICD-10 Code Indicators * (n = 1,686)
Genital	Q50: Congenital malformations of ovaries, fallopian tubes, and broad ligaments	2 (0.12%)
	Q51: Congenital malformations of uterus and cervix	1 (0.06%)
	Q52: Other congenital malformations of female genitalia	23 (1.4%)
	Q53: Undescended and ectopic testicle	173 (10.3%)
	Q54: Hypospadias	364 (21.6%)
	Q55: Other congenital malformations of male genital organs **	607 (36.0%)
	Q56: Indeterminate sex and pseudohermaphroditism	11 (0.65%)
Urinary	Q60: Renal agenesis and other reduction defects of kidney	34 (2.0%)
	Q61: Cystic kidney disease	37 (2.2%)
	Q62: Congenital obstructive defects of renal pelvis and congenital malformations of ureter	243 (14.4%)
	Q63: Other congenital malformations of kidney	113 (6.7%)
	Q64: Other congenital malformations of urinary system	78 (4.6%)

A comprehensive PubMed search was performed using the following search syntax: "genitourinary", "abnormality", "pediatric urology", "maternal drug use", and "pregnancy" for the years 1970-2024.

Several metrics were collected, including the patients' gender, age, race, ethnicity, and payer. The prenatal drug exposures were recorded. The "NAS" diagnosis category was not specific regarding the particular substance used, since it may encompass multiple classes of drugs. The "opioid" diagnosis category explicitly used terms such as "opioids" and "opiates". Therefore, "NAS" and "opioid" were separated as exposure categories in our study. System abnormalities that occurred in conjunction with the GU abnormalities were also documented, including cardiac, circulatory, musculoskeletal, eye/ear/neck/orofacial, nervous system, respiratory, and gastrointestinal/digestive.

Statistical analysis

Descriptive statistics were used to summarize the demographic and clinical characteristics of infants with congenital GU abnormalities associated with maternal drug use. Categorical variables were reported as counts and percentages, while continuous variables were expressed as mean ± standard deviation (SD). Chi-square tests were performed to (1) assess the association between prenatal maternal drug exposure and the occurrence of selected systemic congenital anomalies and to (2) evaluate the association between prenatal maternal drug exposure and the occurrence of selected GU congenital anomalies.

Adjusted multivariate logistic regression models were performed to determine the independent effects of maternal drug exposure while controlling for potential confounders. These models included the (1) primary outcome 1: the presence of any congenital anomaly (yes/no); (2) primary outcome 2: the presence of genitourinary congenital abnormalities (yes/no); and (3) independent variables: infant sex, race, NAS, and maternal exposure to opioids, hallucinogens, cocaine, alcohol, and other noxious substances. All statistical analyses were conducted using SAS version 9.4 (SAS Institute, Cary, NC). A two-sided p-value < 0.05 was considered statistically significant.

## Results

Among 203,518 births over the course of this study, prenatal drug exposures were identified in 9,754 (4.8%) infants, and congenital abnormalities were present in 87,632 (43.1%). Of the infants with congenital abnormalities, 5,406 (6.2%) were prenatally exposed to drugs. Of the 18,467 (9.1%) newborns with GU congenital abnormalities, 1,076 (5.8%) had been prenatally exposed to drugs. The mean age of the 1,076 patients was 6.1 (SD ± 27.1) days at first encounter, and the majority (934, 86.8.0%) of patients were male (Table 2). Most (688, 63.9%) patients were White/Caucasian, and the most common ethnicity was not Hispanic or Latino (1,014, 94.2%). The most frequent payer at first encounter was Medicaid (758, 83.9%).

**Table 2:** Demographics of Patients With Congenital Genitourinary Abnormalities Associated With Prenatal Maternal Drug Use at Our Institution

Metric		Number of Patients (n = 1,076)
Gender	Male	934 (86.8%)
	Female	142 (13.2%)
Primary race	White or Caucasian	688 (63.9%)
	Black or African-American	327 (30.4%)
	Hispanic or Latino	28 (2.6%)
	American Indian or Alaska Native	5 (0.5%)
	Asian	6 (0.6%)
	Other	4 (0.4%)
	Unknown	18 (1.7%)
Ethnicity	Not Hispanic or Latino	1,014 (94.2%)
	Hispanic or Latino	38 (3.4%)
	Unknown	24 (2.2%)
Age (days) at first encounter		Mean (± SD): 6.1 (± 27.1)
Payer at first encounter	Medicaid	758 (83.9%)
	Private/commercial	102 (11.3%)
	Self-pay	37 (4.1%)
	Other government-based	6 (0.7%)
	Medicare	1 (0.1%)

Genitourinary abnormalities

All patients had either genital and/or urinary abnormalities, while 101 (9.4%) had both genital and urinary involvement (Table 3). A total of 792 (73.6%) patients had only genital abnormalities, and 385 (35.8%) had only urinary abnormalities. Of the 1686 total ICD-10 code indicators for GU abnormalities (Q50-56, Q60-64), the most common anomalies associated with prenatal maternal drug use were "other congenital malformations of the male genital organs" (607, 36.0%), followed by "hypospadias" (364, 21.6%) (Table 3).

**Table 3:** Congenital Genitourinary Abnormalities Associated With Prenatal Maternal Drug Use at Our Institution

Genitourinary Abnormalities	Number of Patients (n = 1,076)
Genital Only	792 (73.6%)
Urinary Only	385 (35.8%)
Genital and Urinary	101 (9.4%)
Genital or Urinary	1,076 (100.0%)

There was a statistically significant association between prenatal maternal drug exposure and the occurrence of undescended testis, hypospadias, other congenital malformations of male genital organs, indeterminate sex and pseudohermaphroditism, renal agenesis and other reduction defects of kidney, cystic kidney disease, congenital obstructive defects of renal pelvis and congenital malformations of ureter, other congenital malformations of kidney, and total genitourinary anomalies (all p-values were significant (< 0.05) on chi-square test or Fisher's exact test) (Table 4).

**Table 4:** Congenital Genitourinary Anomalies in Infants at Our Institution From 2014 to 2024, Categorized Based on Prenatal Drug Exposure * Significant (<0.05) p-value of the chi-square test or Fisher's exact test

Variables	Infants		
Selected genitourinary congenital anomaly	No Prenatal Drug Exposure	Prenatal Drug Exposure	P-value
	N = 193,764 (%)	N = 9,754 (%)	
Congenital malformations of ovaries, fallopian tubes and broad ligaments (ICD-10-CM: Q50.*)	47 (0.02%)	2 (0.02%)	1.000
Congenital malformations of uterus and cervix (ICD-10-CM: Q51.*)	41 (0.02%)	1 (0.01%)	0.723
Other congenital malformations of female genitalia (ICD-10-CM: Q52.*)	375 (0.19%)	23 (0.24%)	0.357
Undescended and ectopic testicle (ICD-10-CM: Q53.*)	2,880 (1.49%)	173 (1.77%)	0.023*
Hypospadias (ICD-10-CM: Q54.*)	5,635 (2.91%)	364 (3.73%)	<0.0001*
Other congenital malformations of male genital organs (ICD-10-CM: Q55.*)	10,157 (5.24%)	607 (6.22%)	<0.0001*
Indeterminate sex and pseudohermaphroditism (ICD-10-CM: Q56.*)	95 (0.05%)	11 (0.11%)	0.007*
Renal agenesis and other reduction defects of kidney (ICD-10-CM: Q60.*)	394 (0.20%)	34 (0.35%)	0.002*
Cystic kidney disease (ICD-10-CM: Q61.*)	412 (0.21%)	37 (0.38%)	0.001*
Congenital obstructive defects of renal pelvis and congenital malformations of ureter (ICD-10-CM: Q62.*)	2,984 (1.54%)	243 (2.49%)	<0.0001*
Other congenital malformations of kidney (ICD-10-CM: Q63.*)	1,427 (0.74%)	113 (1.16%)	<0.0001*
Other congenital malformations of urinary system (ICD-10-CM: Q64.*)	1,349 (0.70%)	78 (0.80%)	0.232
Total	17,391 (8.98%)	1,076 (11.03%)	<0.0001*

Prenatal drug exposures

The most common prenatal drug exposures causing GU abnormalities were cannabis (431 patients, 40.1%), followed by NAS/withdrawals (275, 25.6%), opioids (145, 13.5%), and sedatives/hyponotics/anxiolytics (78, 7.3%) (Table 5). Cocaine was detected in 27 (2.5%) patients.

**Table 5:** Prenatal Drug Exposures Causing Genitourinary Abnormalities Associated With Prenatal Maternal Drug Use at Our Institution * The indicator cohort is not comprised of distinct patients; it reflects a sum of all "positive" substance exposure indicators across all distinct patients.

Prenatal Drug Exposures	Values	Cohort Classification	
	Diagnosis Indicator Counts	Exposure Indicators	Entire Patient Cohort
		% of Indicator Cohort (n = 1508) *	% of Entire Cohort (n = 1,076)
Cannabis	431	28.6%	40.1%
Neonatal abstinence syndrome/withdrawals	275	18.2%	25.6%
Other drugs of addiction	171	11.3%	15.9%
Opioids	145	9.6%	13.5%
Other psychoactive substances	103	6.8%	9.6%
Sedatives/hypnotics/anxiolytics	78	5.2%	7.3%
Tobacco	77	5.1%	7.2%
General perinatal drug use and complications	59	3.9%	5.5%
Cocaine	27	1.8%	2.5%
Unspecified drugs of addiction	48	3.2%	4.5%
Alcohol/fetal alcohol syndrome	31	2.1%	2.9%
Other noxious substances	23	1.5%	2.1%
Other maternal medications	23	1.5%	2.1%
Amphetamines and other stimulants	6	0.4%	0.6%
Unspecified maternal medications	7	0.5%	0.7%
Hallucinogens	2	0.1%	0.2%
Anticonvulsants	2	0.1%	0.2%

Infants with NAS were 16% less likely to have GU congenital anomalies compared to those without NAS, after adjusting for sex, race, and maternal exposure to opioids, hallucinogens, cocaine, alcohol, and other noxious substances (adjusted odds ratio (AOR) = 0.84, 95% CI: 0.73-0.96, p = 0.011; Table 6).

**Table 6:** Infant Prenatal Drug Exposures Associated With Congenital Anomalies at Our Institution * Significant p-value (< 0.05); CI = confidence interval; OR = odds ratio

Prenatal Exposure-Related Diagnosis by Drug(s)	Probability of all Infant Congenital Body System Anomaly Diagnoses			Probability of Infant Genitourinary Anomaly Diagnosis			
								Adjusted OR	95% CI	P-value	Adjusted OR	95% CI	P-value	
Neonatal abstinence syndrome	1.45	1.34-1.56	<0.0001*	0.84	0.73-0.96	0.011*	
Opioids	1.74	1.53-1.97	<0.0001*	1.64	1.35-1.99	<0.0001*	
Cocaine	1.25	0.94-1.68	0.131	1.59	1.04-2.44	0.034*	
Alcohol/fetal alcohol syndrome	3.10	2.24-4.27	<0.0001*	1.53	1.01-2.32	0.043*	
Other noxious influence	1.28	1.03-1.59	0.027*	0.68	0.44-1.05	0.084	
Sex (male vs female)	1.43	1.41-1.46	<0.0001*	7.78	7.43-8.15	<0.0001*	
Race (African-American versus Caucasian)	1.37	1.34-1.40	<0.0001*	1.00	0.96-1.04	0.826	
Race (other versus Caucasian)	0.98	0.95-1.00	0.081	0.82	0.78-0.87	<0.0001*	

Infants prenatally exposed to opioids were 64% more likely to have GU congenital anomalies compared to those without opioid exposure, after adjusting for sex, race, NAS, and maternal exposure to hallucinogens, cocaine, alcohol, and other noxious substances (AOR = 1.64, 95% CI: 1.35-1.99, p < 0.0001). Infants prenatally exposed to cocaine were 59% more likely to have GU congenital anomalies compared to those without cocaine exposure, after adjusting for sex, race, NAS, and maternal exposure to opioids, hallucinogens, alcohol, and other noxious substances (AOR = 1.59, 95% CI: 1.04-2.44, p = 0.034). Infants prenatally exposed to alcohol were 53% more likely to have GU congenital anomalies compared to those without alcohol, after adjusting for sex, race, and maternal exposure to opioids, hallucinogens, cocaine, alcohol, and other noxious substances (AOR = 1.53, 95% CI: 1.01-2.32, p = 0.043). Males were 7.78 times more likely to have GU congenital anomalies than females, after adjusting for race, NAS, and maternal exposure to opioids, hallucinogens, cocaine, alcohol, and other harmful substances (AOR = 7.78, 95% CI: 7.43-8.15, p < .0001). African-American infants were 18% less likely to have GU congenital anomalies compared to Caucasian infants, after adjusting for sex, NAS, and maternal exposure to opioids, hallucinogens, cocaine, alcohol, and other noxious substances (AOR = 0.82, 95% CI: 0.78-0.87, p < 0.0001).

Although the two variables (NAS and opioid exposure) were associated, they were not redundant: 18.1% of patients with NAS were also coded for opioid exposure, while 50.0% of patients with opioid exposure had NAS (phi = 0.295). A sensitivity analysis was performed by refitting the multivariable model after excluding NAS and opioid exposure separately. The adjusted estimates for the remaining covariates were essentially unchanged. The adjusted OR for opioid exposure changed from 1.64 (95% CI: 1.35-1.99) to 1.51 (95% CI: 1.26-1.81) after excluding NAS, while the adjusted OR for NAS changed from 0.84 (95% CI: 0.73-0.96) to 0.92 (95% CI: 0.81-1.05) after excluding opioid exposure, an attenuation consistent with the clinical expectation that part of the NAS association operates through opioid exposure. These findings suggest some expected overlap but do not indicate substantial instability from including both variables in the model.

Concurrent genitourinary abnormalities with other congenital system abnormalities

In addition to the GU abnormalities due to maternal drug use, 363 (33.7%) patients had cardiac congenital abnormalities, 278 (25.8%) had circulatory abnormalities, and 268 (24.9%) had musculoskeletal abnormalities (Table 7). There was a statistically significant association between prenatal maternal drug use and the occurrence of congenital abnormalities of all body systems (< 0.0001) (Table 8).

**Table 7:** Congenital Body System Anomaly Co-occurrence With Genitourinary Abnormalities Associated With Maternal Drug Use at Our Institution

Body System	Values	Cohort Classification	
	Diagnosis Indicator Counts	Patients with ≥ 1 Other System Diagnosis	Entire Patient Cohort
		% of Sub-cohort (n=717)	% of Entire Cohort (n=1,076)
Cardiac	363	50.6%	33.7%
Circulatory	278	38.8%	25.8%
Musculoskeletal	268	37.4%	24.9%
Eye/ear/neck/orofacial	180	25.1%	16.7%
Nervous system	152	21.2%	14.1%
Respiratory	110	15.3%	10.2%
Gastrointestinal/digestive	59	8.2%	5.5%
Other	375	52.3%	34.9%
Chromosomal	78	10.9%	7.3%

**Table 8:** Congenital Anomalies of All Body Systems at Our Institution, Categorized Based on Prenatal Drug Exposure ^a^ P-value of the chi-Square test

Body System	Infants		
	No Prenatal Drug Exposure	Prenatal Drug Exposure	P-value^a^
	N = 193,764 (%)	N = 9,754 (%)	
Cardiac	21,816 (11.3%)	2,081 (21.3%)	<0.0001
Circulatory	13,306 (6.9%)	1,376 (14.1%)	<0.0001
Musculoskeletal	21,937 (11.3%)	1,463 (15.0%)	<0.0001
Eye/ear/neck/orofacial	16,376 (8.5%)	1,037 (10.6%)	<0.0001
Nervous system	5,630 (2.9%)	588 (6.0%)	<0.0001
Respiratory	2,945 (1.5%)	353 (3.6%)	<0.0001
Gastrointestinal/digestive	1,959 (1.0%)	209 (2.1%)	<0.0001
Other	28,482 (14.7%)	2,234 (22.9%)	<0.0001
Chromosomal	1,882 (1.0%)	224 (2.3%)	<0.0001
Genital	13,402 (6.9%)	792 (8.1%)	<0.0001
Urinary	5,219 (2.7%)	385 (4.0%)	<0.0001
Total	82,226 (42.4%)	5,406 (55.4%)	<0.0001

## Discussion

A paucity of studies have addressed the use of maternal drugs during pregnancy and the association with genital/urinary abnormalities [11-18]. In Bunn et al.'s study of congenital malformations associated with prenatal exposure to drugs in Kentucky in newborns (at birth) and infants (after birth up to 30 days old) from 2009 to 2013, congenital anomalies were diagnosed in 11.2% of newborns prenatally exposed to drugs compared with 8.5% of those with no prenatal drug exposure [13]. Of the newborns with prenatal drug exposure-related diagnoses, 55% were male, 93% were Caucasian, and 81% had Medicaid. GU anomalies were significantly elevated among newborns with prenatal drug exposures, and genital anomalies were significantly increased among infants [13]. NAS was the most common prenatal drug exposure-related diagnosis in newborns. Newborns and infants diagnosed with congenital malformations (cardiac/circulatory/genital/urinary/integument) following prenatal exposure to drugs had significantly extended hospitalization stays and higher total hospitalization charges compared to those who were not prenatally exposed to drugs [13]. These authors also posit that prenatal exposure to polydrugs may have a higher likelihood of severe anomalies in newborns and infants compared with prenatal exposure to a single drug [13].

Our study concurs with Bunn et al.'s study with respect to the association between prenatal drug exposure and GU abnormalities, as well as the higher percentage of males, Caucasians, and Medicaid coverage. Hypospadias was diagnosed in 22% of the GU ICD-10 code indicators in our study. It should be noted that the large sex difference between boys and girls in our study may be a labeling artifact and not a real biological finding. Conditions such as hypospadias and undescended testis can only be diagnosed in boys. Therefore, boys will always show more "male genital" diagnosis codes, not necessarily because they are biologically more at risk. Bunn et al. did not specify the particular GU anomaly associated with prenatal maternal drug use [13]. NAS was the most frequent prenatal drug exposure-related diagnosis in newborns in Bunn et al.'s work, whereas it was the second most common exposure in the current study following cannabis exposure. In our study, infants prenatally exposed to opioids, cocaine, or alcohol were 64%, 59%, and 53%, respectively, more likely to have GU congenital anomalies compared to those without prenatal maternal drug use.

Few publications have reported the use of cocaine in causing GU anomalies [11,12,14]. In Chavez et al.'s case-control study of liveborn and stillborn infants (276 with urinary anomalies and 791 anomalies), a significant association was observed between reported cocaine use (between one month prior to pregnancy through the first three months of pregnancy) and an increased risk for urinary tract defects [14]. The three exposed neonates had the following urinary tract defects: (1) bilateral congenital hydronephrosis; (2) incompetent ureteral orifices causing congenital hydronephrosis; and (3) unilateral renal and ureteral agenesis. Although no statistically significant association was demonstrated between reported cocaine use and genital defects, the genital abnormalities affected four patients (hypospadias in two patients, hypospadias and congenital chordee in one, and bifid scrotum and imperforate anus with a rectovesical fistula in one). In Battin et al.'s study of 136 neonates with a positive cocaine history or urine drug screen, 79 underwent a renal ultrasound (US) [11]. Abnormal findings on the US were detected in 11 (14%) of these 79 patients, including renal tract dilation (eight patients), horseshoe kidney (two patients), unilateral abnormal small kidney (one patient), and duplex kidney (one patient). Clinical findings consisted of glandular (two patients) and juxtaglandular (one patient) hypospadias with chordee; these patients did not have an abnormal US. These authors suggested that routine US screening may be necessary for all infants exposed to cocaine in utero [11]. In Bauer et al.'s study of 717 cocaine-exposed neonates and 7,442 nonexposed neonates, hypospadias was diagnosed in four (0.6%) patients in the former group, and 23 (0.3%) in the nonexposed group [12].

Several other drugs taken during pregnancy are associated with GU abnormalities, including antileptic medications [16], macrolides [15], buprenorphine or methadone [18], fentanyl [19], all opioids [8], diethylstilbestrol [20,21], and zidovudine [17]. Antiepileptic medications (most commonly with valproate in a dose-dependent manner) may be associated with absent kidney, duplicate renal pelvis, hypospadias, hydronephrosis, extra renal pelvis, and undescended testicle [16]. In Fan et al.'s study of mothers prescribed a macrolide (erythromycin, clarithromycin, or azithromycin) monotherapy or one penicillin monotherapy from the fourth gestational week to delivery, macrolide prescribing in any trimester was associated with an increased risk of genital malformations, primary hypospadias (4.75 vs 3.07 per 1,000) [15]. In Rough et al.'s study of pregnant women with HIV, the odds of male genital malformations were more than doubled for women treated with zidovudine in the first trimester compared to those treated with antiretroviral therapy without zidovudine [17]. Offspring of women who took diethylstilbestrol during pregnancy have an increased incidence of genital abnormalities (epididymal cysts, undescended testes, small testes in males and structural reproductive tract anomalies in females) and an elevated risk of prostate and testicular cancer [20,21]. A novel syndrome associated with prenatal fentanyl exposure has also been reported, which consists of microcephaly, short stature, distinctive facial features, cleft palate, talipes equinovarus or rocker bottom feet, and chordee or hypospadias [19].

Strengths and limitations

The strength of the present study is that it highlights a large number of patients with GU abnormalities in infants exposed to maternal drug use. There is a high number of prenatal drug use in our state, which allows us to evaluate any associations with GU abnormalities. Our 11-year study emphasizes the importance of recognizing GU anomalies, especially in patients who have concurrent congenital cardiac/circulatory/musculoskeletal system abnormalities. It should also be emphasized that not all drugs taken during gestation are illegal; some drugs are prescribed during pregnancy to treat different illnesses. The limitations of the present study include its retrospective nature and the fact that the data were obtained from only our institution. Additionally, not all women report drug use during pregnancy, so the actual rates may be higher. We were also unaware of the timing of drug exposure during pregnancy in terms of specific trimester and the amount that was consumed. The results suggest that the two variables (NAS and opioid exposure) are related but not redundant, and the model estimates were generally stable when either variable was removed. However, this potential overlap is a limitation of our study. Additional limitations of the current study are the heterogeneity of the included studies, potential confounding and selection bias, and the limited ability to establish causation.

## Conclusions

Prenatal drug exposure is a public health dilemma and adversely affects several neonatal body systems. Certain drugs during pregnancy may be associated with GU abnormalities in the neonate, such as opiates, cannabinoids, tobacco, alcohol, and psychostimulants. In this observational study, we sought to determine the incidence and characteristics of GU abnormalities in infants who were exposed to maternal drug use during pregnancy. Based on the heterogeneity of the available studies, physicians, parents, and other caregivers should be aware of the association between GU abnormalities and prenatal drug use. Pregnant women should also be educated about the potential for GU abnormalities. It is important to emphasize the need for prenatal imaging to rule out such abnormalities. Further research is imperative to assess the specific drugs and toxicities associated with GU abnormalities following prenatal drug exposure.
